# Conventional Versus Accelerated Ponseti in the Management of Cases of Idiopathic Clubfoot: A Systematic Review and Meta-Analysis

**DOI:** 10.7759/cureus.45041

**Published:** 2023-09-11

**Authors:** Mokhtar A Alsayed, Mohamed A Hussein, Raad M Althaqafi, Ali Alyami

**Affiliations:** 1 Orthopedic Surgery, Armed Forces Hospital, Taif, SAU; 2 Pediatric Orthopedic Surgery, National Institute of Neuromotor System, Giza, EGY; 3 Orthopedic Surgery, King Abdulaziz Specialist Hospital, Taif, SAU; 4 Muscloskeletal Oncology, Limb Reconstructive Surgery, Sport Medicine and Arthroscopy, King Saud bin Abdulaziz University for Health Sciences, King Abdul Aziz Medical City, Jeddah, SAU

**Keywords:** prospective comparative design, randamized trial, congenital talipes equinovarus deformity, idiopathic clubfoot, ponseti's method

## Abstract

This study aimed to compare the outcomes of the accelerated and standard Ponseti method for clubfoot pathology by constructing a systematic review and meta-analysis of relevant randomized controlled trials and nonrandomized comparative studies. A systematic search was conducted to identify the relevant studies through PubMed, Google Scholar, and Cochrane depending on Preferred Reporting Items for Systematic Reviews and Meta-Analyses (PRISMA) guidelines. The keywords used included “accelerated” AND “standard” AND “Ponseti” AND “clubfoot” AND “Congenital Talipes Equinovarus” AND “CTEV” AND “prospective comparative design” AND “randomized trial.”

We conducted this analysis among 13 studies that met the criteria adopted in this review where eight studies were prospective comparative studies, and five studies were randomized prospective comparative studies which were published in the period between 2015 and 2022. Statistically, accelerated Ponseti showed superior impact over standard Ponseti considering the duration of treatment (22.53 days vs. 40.61 days, p<0.001). No significant difference was reported between the two methods considering final Pirani score (0.64 vs. 0.56, p=0.194), number of casts (5.23 vs. 5.25, p=0.425), rate of tenotomy (66.2% vs. 63.1%, OR=1.246, 95% CI: 0.86-1.80, p=0.245), relapse rate (9.51% vs. 8.54%, OR=1.126, 95% CI: 0.68-1.86, p=0.642) and complication rate (14.4% vs. 13.1%, OR=1.130, 95% CI: 0.58-2.19, p=0.717). We concluded that the accelerated Ponseti method could achieve comparable efficacy to the standard method in terms of post-procedure Pirani score, tenotomy rate, relapse rate, complications rate, and number of casts needed by the patients with advantage of requiring shorter duration of treatment which is associated with more patient’s compliance.

## Introduction and background

Clubfoot, one of the most congenital deformities, also known as Congenital Talipes Equinovarus (CTEV), affects about 1-6.8 for every 1000 live births [1-3]. It consists of four components, including ankle equinus, forefoot adductus, hindfoot varus, and midfoot cavus [4,5]. If left untreated, this could lead to stiffness, weakness, and chronic pain, permanent disability in the absence of a series of revision surgeries [6]. Therefore, early diagnosis and holistic care are significant factors in the successful management of patients with CTEV [7].

The first advanced nonoperative treatment of clubfoot was plaster of Paris casts which was introduced by Guerin in 1836 [7]. Surgical intervention at this time was the main management of clubfoot because it was believed that it was necessary to achieve the best therapeutic outcomes. However, the long-term follow-up-based evidence showed disappointing clinical, radiographic, and kinematic outcomes in patients treated with surgical intervention. Moreover, corrective surgery when conducted in infants often results in adolescent pain, decreased strength, and functional deficits [8]. Furthermore, surgical procedures including repeated soft tissue releases on the clubfoot induce some complications such as stiffness of foot, arthritis disorders, and poor quality of life [9]. A number of conservative techniques have been proposed to correct clubfoot deformity with the following techniques: different methods of manipulations, orthosis, splinting or bracing, and casting and strapping [10,11]. One such conservative technique was kite technique which was introduced in 1939, and included manipulation and casting techniques; however, its associated success rate was poor [12]. In the 1940s, Dr. Ignacio Ponseti presented Ponseti casting as a conservative approach to clubfoot based on the fundamental pathoanatomic and kinematics of the deformity [13]. Ponseti published the results of his method in 1963, providing evidence that it yielded satisfactory outcomes in 90% of the patients. This method uses Achilles tenotomy to release the equinus deformity and brace the corrected clubfoot [14,15], which helps in obtaining a plantigrade, functional, and pain-free foot [16]. This method has the advantage of being applied to patients as early as one day old and has been proven to realign the clubfoot in infants while avoiding extensive and major surgeries [13]. For these reasons, weekly corrective manipulation and long-leg casting have been chosen as the standard care of management in the modern era, as the best option to gradually correct all symptoms associated with clubfoot deformity [1,6,7]. However, despite the huge advantages of the Ponseti casting considering cost-effectiveness and safety, it has some limitations mainly the long time it takes to correct all deformity components [6,17]. This management makes compliance a challenge for patients with limited economic resources and difficulty accessing care [18]. Some literature has investigated the efficacy of accelerated Ponseti casting where the plasters are applied identically to the standard protocol; however, changed more frequently (two times weekly). Additionally, few studies have reviewed the difference between those interventions. Therefore, this study aimed to compare the outcomes of the accelerated and standard Ponseti method for clubfoot pathology.

## Review

Methodology

**Figure 1:**
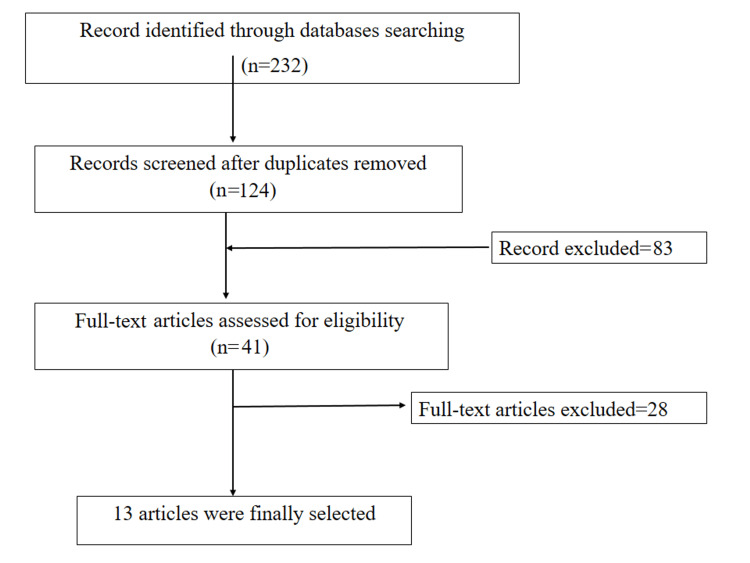
Flowchart showing article selection based on PRISMA guidelines. The image summarizes Preferred Reporting Items for Systematic Reviews and Meta-Analyses (PRISMA) steps to select the valid article to be included in this systematic review.

This is a systematic review and meta-analysis of relevant randomized controlled trials and nonrandomized comparative studies. A systematic search was conducted to identify the relevant studies through PubMed and Cochrane depending on PRISMA guidelines (Figure 1). The keywords used included “accelerated” AND “standard” AND “Ponseti” AND “clubfoot” AND “Congenital Talipes Equinovarus” AND “CTEV” AND “prospective comparative design” AND “randomized trial.”

The inclusion criteria of studies included in this review are as follows: all studies published between 2015 and 2022 that compare accelerated and standard Ponseti methods; studies including pediatric populations under the age of 10 years with diagnosis of CTEV/clubfoot; studies including at least one of the following outcomes: pre- and post-Pirani score, number of casts needed, duration of treatment, relapse rate, tenotomy rate and/or complication rate; studies published in English or other languages; however, only English version was included; studies depending on randomized controlled trial or prospective cohort comparative designs published between 2015 and 2022. The exclusion criteria of the included studies are as follows: studies that reported the presence of comorbid infectious diseases or malignancy; studies depending on surgical treatments or conservative approaches rather than accelerated and standard Ponseti; non-comparative study designs; studies depending on non-human in vivo or in vitro design; study with retrospective/systematic review design, review articles, books, unpublished articles; studies published in non-English language without an English version; and those published before 2015. Table 1 shows the inclusion and exclusion criteria according to the Population, Intervention, Comparison, and Outcome (PICO) method.

**Table 1:** Inclusion and exclusion criteria according to the Population, Intervention, Comparison, and Outcome (PICO) method. The implemented criteria for inclusion or exclusion of studies and considering its validity for this systematic review.

Study component	Inclusion	Exclusion
Population	(1) ≤10 years of age at initial treatment. (2) Clinical diagnosis of CTEV	(1) >10 years of age at initial treatment. (2) Less than two months of follow-up. (3) Comorbid infection or malignancy. (4) Animal studies
Intervention and comparison	(1) Accelerated and standard Ponseti methods (comparison)	(1) Surgical intervention. (2) All other conservative treatments
Outcome	(1) Pirani score (pre- and post-procedure), number of casts needed, duration of treatment, tenotomy rate, relapse rate, complication rate	(1) No outcome mentioned or different outcomes
Publication	(1) Primary research published in English in a journal. (2) Published in the period between 2015 and 2022	(1) Abstracts, editorials, letters. (2) Conference presentations or proceedings. (3) Books. (4) Duplicate publications of the same study/cohort that do not report on different outcomes
Design	(1) Randomized controlled trials. (2) Prospective cohort studies	(1) Case reports or series. (2) Review articles. (3) Retrospective studies

After the selection of the qualified studies, specific data were extracted from each study regarding characteristics such as year of publication, setting, age of patients, sex of patients, number of patients, number of feet, type of deformities (bilateral or unilateral), follow-up duration, and Pirani score for patients of each method (Accelerated or standard). Moreover, data regarding outcomes were extracted, including post-procedure Pirani score, number of casts needed, duration of treatment, tenotomy rate, relapse rate, and complication rate. Continuous variables. including post-procedure Pirani score, number of casts needed, and duration of treatment were compared in terms of weighted mean difference (WMD), whereas dichotomous variables including tenotomy rate, complications rate, and relapse rate, were assessed in terms of odds ratio (OR) and 95% confidence intervals (CI). A fixed-effect model was used when heterogeneity (I^2^) was <50%, whereas a random-effect model was used when it was >50%. Statements with p-values lower than 0.05 were considered significant.

Results

The electronic search strategy conducted in this review ended in 232 hits which after removing duplicates reduced to 124 studies. These 124 studies were considered eligible for further evaluation, from which 83 studies were excluded based on title and abstract resulting in 41 studies. Among these studies, 28 were excluded according to the inclusion and exclusion criteria. Therefore, the final number of studies included in the current retrospective study was 13 studies (Figure 1).

All studies listed in Table 2 were conducted between 2011 and 2022. Among these 13 included studies listed in Table 2, eight studies (nos. 1-5,7,10,11) were prospective comparative studies while five studies (nos. 6,8,9,12,13) were randomized prospective comparative studies. Most of the included studies in Table 2 (nos. 1,2,4-9,13) were conducted in India, Egypt (no. 3), Iraq (no. 10), Nigeria (no. 11), and Pakistan (no. 12); however, no study having the inclusion criteria was found to be conducted in Europe region (Table 2).

**Table 2:** Studies included in the analysis. The table includes the authors' first name, year of publication, journal name, country where the study was conducted, and the study design of each of the included studies.

S. no.	Studies	Year	Journal	Country	Study design
1	Barik et al. [1]	2019	European Journal of Orthopaedic Surgery and Traumatology	India	Prospective comparative study
2	Solanki et al. [7]	2018	Journal of Orthopaedics, Traumatology and Rehabilitation	India	Prospective comparative study
3	Elgohary and Abulsaad [17]	2015	European Journal of Orthopaedic Surgery and Traumatology	Egypt	Prospective comparative study
4	Mageshwaran et al. [18]	2016	International Journal of Scientific Study	India	Prospective comparative study
5	Singh et al. [19]	2021	International Journal of Research in Medical Sciences	India	Prospective comparative study
6	Sharma et al. [20]	2018	Orthopaedic Journal of M P Chapter	India	Randomized prospective comparative study
7	Kumar and Singh [21]	2020	European Journal of Molecular and Clinical Medicine	India	Prospective comparative study
8	Islam et al. [22]	2020	Clinics in Orthopedic Surgery	India	Randomized prospective comparative study
9	Dutta et al. [23]	2019	International Journal of Orthopaedics Sciences	India	Randomized prospective comparative study
10	Doski and Jamal [24]	2021	Zanco Journal of Medical Sciences	Iraq	Prospective comparative study
11	Anikwe et al. [25]	2021	International Journal of Paediatric Orthopaedics	Nigeria	Prospective comparative study
12	Ahmed et al. [26]	2019	Journal of Pakistan Orthopaedic Association	Pakistan	Randomized prospective comparative study
13	Ahmad et al. [27]	2020	Journal of Medical Science and Clinical Research	India	Randomized prospective comparative study

The study included 13 studies and a pooled sample size of 701 patients with 929 affected feet and mean age of 72.8 days (2.43 months). Moreover, 60.2% of the patients were males (n=422), whereas females represented 39.8% (n=279) with male to female ratio of 1.51:1. Across the studies, 337 patients (469 feet) were randomized in the standard Ponseti group with mean age of 80.775 days (2.69 months), and 62.2% were males. In the accelerated Ponseti group, 333 patients (460 feet) were included with mean age of 64.8 days (2.16 months); 56.66% were males and 31 patients with no reported information of their randomization in one study as the authors only reported the total number of patients and did not show the number of patients in each group [7]. Moreover, among 621 patients, 48.1% had bilateral affected feet, whereas 51.9% had unilateral feet with no difference between groups (bilateral represented 48.5% of 313 patients and 47.7% of 308 patients with standard and accelerated groups, respectively) (p=0.492) (Tables 3, 4).

**Table 3:** The sample size of patients, the sample size of feet, and the follow-up duration of patients for each included study.

Studies		Sample size (patients)			Sample size (feet)			Age (days)		
		Total	Standard	Accelerated	Total	Standard	Accelerated	Total	Standard	Accelerated
1	Barik et al. [1]	30	15	15	51	26	25	1	12.35	9.84
2	Solanki et al. [7]	31	NA	NA	40	20	20	5	141.3	81
3	Elgohary and Abulsaad ​​​​​​[17]	41	20	21	66	34	32	3	74.9	80.99
4	Mageshwaran et al. [18]	40	20	20	51	26	25	2	28.4	28.1
5	Singh et al. [19]	40	21	19	61	31	30	3	29	21
6	Sharma et al. [20]	40	20	20	53	26	27	1	22.95	23.54
7	Kumar and Singh [21]	70	35	35	100	50	50	1	24.9	27.5
8	Islam et al. [22]	100	50	50	158	81	77	6	29.2	18.2
9	Dutta et al. [23]	64	32	32	100	48	52	10	86.7	102.6
10	Doski and Jamal [24]	48	23	25	79	39	40	6	NA	NA
11	Anikwe et al. [25]	62	34	28	90	48	42	36	354	243
12	Ahmed et al. [26]	80	40	40	NA	NA	NA	2	48	47.4
13	Ahmad et al. [27]	55	27	28	80	40	40	4	117.6	94.5

**Table 4:** The demographic distribution of the included patient's sex and foot-affected side for each study.

Studies		Sex												Side			
		Total				Standard				Accelerated				Standard		Accelerated	
		Male	%	Female	%	Male	%	Female	%	Male	%	Female	%	Bilateral	Unilateral	Bilateral	Unilateral
1	Barik et al. [1]	22	73.3	8	26.7	NA	NA	NA	NA	NA	NA	NA	NA	11	-	-	-
2	Solanki et al. [7]	19	61.3	12	38.7	NA	NA	NA	NA	NA	NA	NA	NA	NA	-	-	-
3	Elgohary and Abulsaad [17]	26	63.4	15	36.6	14	70.0	6	30.0	12	57.1	9	42.9	14	-	-	-
4	Mageshwaran et al. [18]	20	50.0	20	50.0	12	60.0	8	40.0	11	55.0	9	45.0	6	-	-	-
5	Singh et al. [19]	22	55.0	18	45.0	12	57.1	9	42.9	10	52.6	9	47.4	10	-	-	-
6	Sharma et al. [20]	20	50.0	20	50.0	8	40.0	12	60.0	12	60.0	8	40.0	6	-	-	-
7	Kumar and Singh [21]	40	57.1	30	42.9	22	62.9	13	37.1	18	51.4	17	48.6	15	-	-	-
8	Islam et al. [22]	70	70.0	30	30.0	36	72.0	14	28.0	34	68.0	16	32.0	31	-	-	-
9	Dutta et al. [23]	36	56.3	28	43.8	19	59.4	13	40.6	17	53.1	15	46.9	16	-	-	-
10	Doski and Jamal [24]	32	0.7	16	0.3	NA	NA	NA	NA	NA	NA	NA	NA	16	-	-	-
11	Anikwe et al. [25]	39	62.9	23	37.1	21	61.8	13	38.2	18	64.3	10	35.7	14	20	14	14
12	Ahmed et al. [26]	42	52.5	38	47.5	24	60.0	16	40.0	18	45.0	22	55.0	NA	11	11	8
13	Ahmad et al. [27]	34	61.8	21	38.2	18	66.7	9	33.3	16	57.1	12	42.9	13	14	12	16

In Table 5, we showed the results of each study considering the outcomes of the use of both standard and accelerated Ponseti. The mean follow-up duration of patients with standard Ponseti was 8.23 months ranging between two and 25.5 months, while among patients with accelerated Ponseti, the follow-up duration was 8.04 months ranging between two and 23.38 months. Pre-procedure Pirani collected from each study showed mean scores of 4.99 (SD=0.32) and 5.08 (SD=0.42) in standard and accelerated groups, respectively, (no significant difference in the baseline Pirani score, p=0.551). After the operation, the Pirani score was significantly decreased in both operations to 0.46 (SD=0.36) in standard Ponseti and 0.53 (SD=0.46) in accelerated one without a significant difference between them.

**Table 5:** Analyzed outcomes of the studies. The follow-up period (month), pre-procedure Pirani score, post-procedure Pirani score, number of casts needed, duration of treatment (days), tenotomy rate, relapse rate, and complications are presented for each study.

S. no.	Studies	Follow-up period (month)		Pre-procedure Pirani score		Post-procedure Pirani score		Number of casts needed		Duration of treatment (days)		Tenotomy rate		Relapse rate		Complications (pressure sores)	
		Standard	Accelerated	Standard	Accelerated	Standard	Accelerated	Standard	Accelerated	Standard	Accelerated	Standard	Accelerated	Standard	Accelerated	Standard	Accelerated
1	Barik et al. [1]	2	2	5.02	5.02	1.25	1.5	5.23	4.72	54.38	33.88	42.31%	52.00%	NA	NA	NA	NA
2	Solanki et al. [7]	3	3	4.6	5.35	0.525	0.5	6.35	7	47.25	18.45	55.00%	65.00%	NA	NA	NA	NA
3	Elgohary and Abulsaad [17]	25.25	23.38	5.17	5.13	0.49	0.52	4.88	5.16	33.36	18.13	91.18%	93.75%	14.71%	15.63%	NA	NA
4	Mageshwaran et al. [18]	6	6	4.97	5.025	0.075	0.1	5.55	5.95	52.8	39.65	11.54%	24.00%	11.54%	16%	NA	NA
5	Singh et al. [19]	12	12	5	5.5	0	0	6.35	6	47.25	18	85.71%	89.47%	14.29%	15.79%	NA	NA
6	Sharma et al. [20]	7.7	8.26	5.32	5.21	0.4	0.23	4.92	4.98	35.24	14.19	0.00%	0.00%	0.00%	0.00%	0.00%	0.00%
7	Kumar and Singh [21]	3	3	4.91	5.42	0.08	0.14	5.77	6.12	52.8	41.11	11.43%	25.71%	17.14%	25.71%	NA	NA
8	Islam et al. [22]	12	12	4.67	4.35	0.34	0.35	6.1	5.77	39.5	52.8	86.42%	84.42%	3.70%	2.60%	23%	25.97%
9	Dutta et al. [23]	6	5	5.25	5.37	0.42	0.44	5.92	6.09	41.42	21.13	85.42%	88.46%	6.25%	5.77%	NA	NA
10	Doski and Jamal [24]	6	6	5.6	5.57	0.47	0.77	5.09	5.82	35.63	19.37	94.87%	97.50%	12.82%	10%	2.56%	5.00%
11	Anikwe et al. [25]	3	3	4.455	4.325	1	1.32	4.25	3.875	29.75	13.3	41.70%	28.57%	2.08%	2.38%	4.17%	2.38%
12	Ahmed et al. [26]	6	6	NA	NA	NA	NA	NA	NA	36.88	20.73	NA	NA	NA	NA	NA	NA
13	Ahmad et al. [27]	15	15	4.88	4.69	0.5	0.48	5.14	6.53	40.6	15.47	85%	92.50%	12.50%	15%	0%	0%

**Figure 2:**
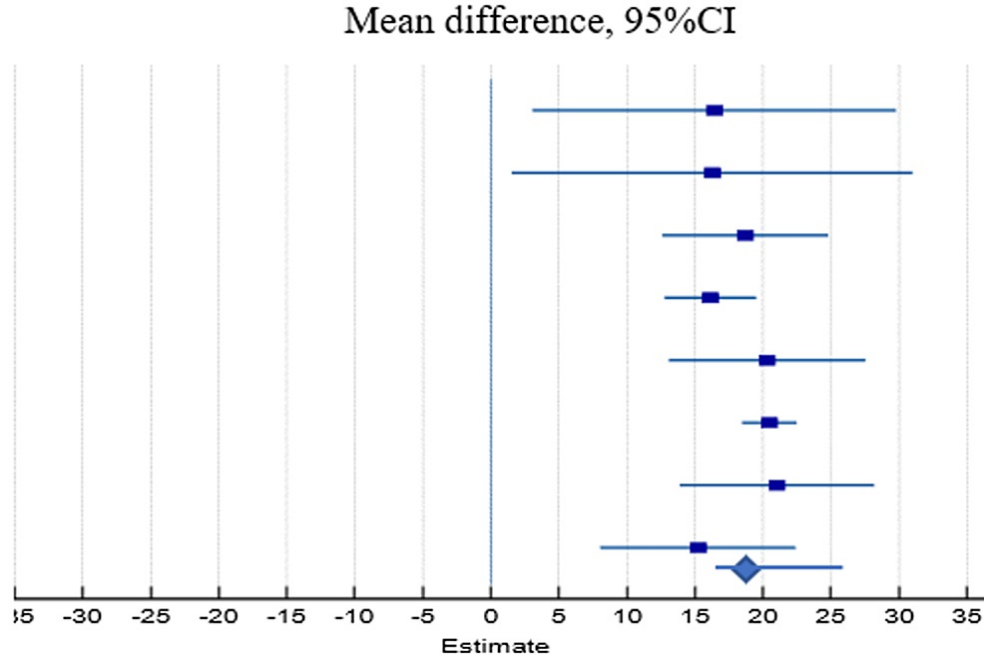
Forest plot analysis of the duration of the treatment for each patient in the included studies.

Of the six outcomes analyzed, a meta-analysis was conducted among the study, where eight studies were included to assess the difference between standard and accelerated methods considering mean duration of treatment. Statistically, accelerated Ponseti showed superior impact over standard Ponseti considering the duration of treatment (22.53 days vs. 40.61 days, p<0.001). The accelerated method had a mean of 19.2 days lower than the standard Ponseti method of treatment (95%: 17.6-20.6) (Table 6, Figure 2).

**Table 6:** Forest analysis of the duration of the treatment in each of the patients in the included studies. Heterogeneity: chi^2^=6.66, df=7 (p=0.464)

Studies	Standard			Accelerated			Weight (%)	Mean difference (95% CI)
	Mean	SD	Total	Mean	SD	Total		
Anikwe et al. [25]	29.75	13.03	34	13.3	6.21	28	1.29	16.5 (3.0-29.8)
Doski and Jamal [24]	35.63	5.1	23	19.37	12.6	25	1.06	16.3 (1.5-30.9)
Islam et al. [22]	58.2	8.3	50	39.5	5.2	50	6.23	18.7 (12.6-24.6)
Ahmed et al. [26]	36.88	5.11	40	20.73	3.4	40	20.48	16.2 (12.7-19.5)
Dutta et al. [23]	41.42	7.62	32	21.13	3.94	32	4.42	20.3 (13.0-27.5)
Barik et al. [1]	54.38	8.01	15	33.88	9.03	15	57.55	20.5 (18.5-22.4)
Sharma et al. [20]	35.24	5.84	20	14.19	2.2	20	4.52	21.1 (13.9-28.1)
Elgohary and Abulsaad [17]	33.36	6.69	20	18.13	3.02	21	4.45	15.2 (8.0-22.4)
Total	40.61	7.46	234	22.53	5.7	231	P≤0.001	19.2 (17.6-20.6)

**Figure 3:**
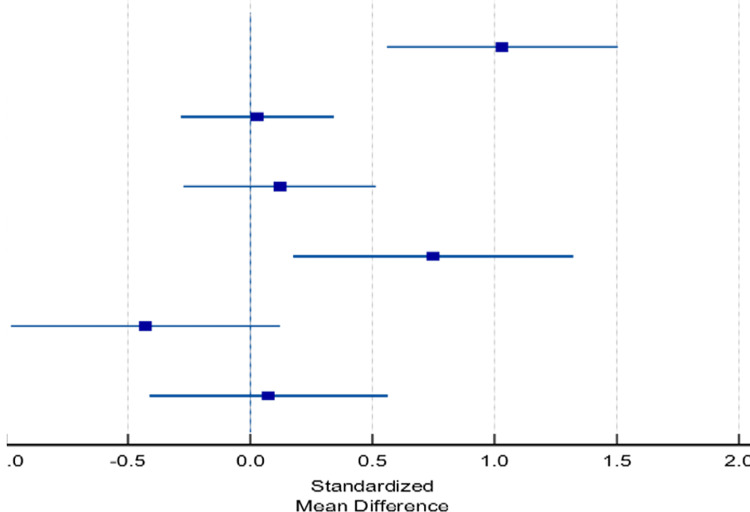
Forest plot analysis for duration of treatment. The plot representation of the analyzed difference between the standard and accelerated Ponseti methods is based on the final Pirani score.

**Figure 4:**
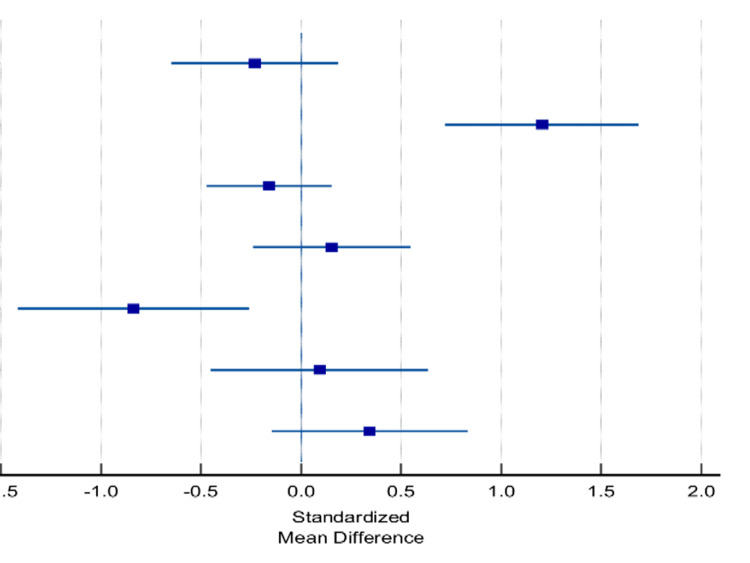
Forest plot for the analyzed count of casts. A plot representation of the analyzed number of casts between the two groups underwent the standard and accelerated Ponseti methods considering the mean duration of treatment.

Furthermore, six studies were included to assess the difference between the two methods in final Pirani score and showed no significant difference (0.64 vs. 0.56, p=0.194; Table 7 and Figure 3). Moreover, six studies were included in the meta-analysis of the number of casts, and no significant difference was found between the number of casts in the standard group and accelerated group (5.23 vs. 5.25, p=0.425; Table 8 and Figure 4).

**Table 7:** Forest analysis for post-procedure Pirani score. SMD: standardized mean difference Analysis of the differences between the standard and accelerated Ponseti methods based on the final Pirani score. Heterogeneity: chi^2^=22.76, df=5 (p=0.004).

Studies	Standard			Accelerated			Weight %	SMD
	Mean	SD	Total	Mean	SD	Total		
Doski and Jamal [24]	0.47	0.41	39	0.77	0.01	40	14.15	1.03 (0.56-1.50)
Islam et al. [22]	0.34	0.38	81	0.35	0.31	77	31.79	0.028 (-0.28-0.34)
Dutta et al. [23]	0.42	0.17	48	0.44	0.16	52	20.18	0.12 (-0.27-0.515)
Barik et al. [1]	1.25	0.46	26	1.5	0	25	9.78	0.749 (0.17-1.32)
Sharma et al. [20]	0.4	0.43	26	0.23	0.35	27	10.64	-0.428 (-0.97-0.122)
Elgohary and Abulsaad [17]	0.49	0.42	34	0.52	0.38	32	13.46	0.07 (-0.41-0.56)
Total	0.56	0.38	254	0.64	0.20	253	P=0.194	0.21 (0.04-0.39)

**Table 8:** Forest analysis for count of casts between standard and accelerated Ponseti methods. Meta-analysis of number of casts between the two groups underwent the standard and accelerated Ponseti methods considering mean duration of treatment. SMD: standardized mean difference

Studies	Standard			Accelerated				SMD
	Mean	SD	Total	Mean	SD	Total	Weight	
Anikwe et al. [25]	4.25	1.675	48	3.875	1.53	42	15.4	-0.231 (-0.64-0.18)
Doski and Jamal [24]	5.09	0.59	39	5.82	0.61	40	11.57	1.204 (0.72-1.68)
Islam et al. [22]	6.3	1.1	81	6.1	1.4	77	27.04	-0.159 (-0.47-0.155)
Dutta et al. [23]	5.92	1.09	48	6.09	1.11	52	17.2	0.153 (-0.24-0.55)
Barik et al. [1]	5.23	0.59	26	4.72	0.61	25	8.21	-0.83 (-1.42—0.25)
Sharma et al. [20]	4.92	0.77	26	4.98	0.49	27	9.28	0.09 (-0.45-0.63)
Elgohary and Abulsaad [17]	4.88	0.88	34	5.16	0.72	32	11.32	0.343 (-0.15-0.83)
Total	5.23	0.96	302	5.25	0.92	295	P=0.425	0.065 (-0.096-0.228)

**Figure 5:**
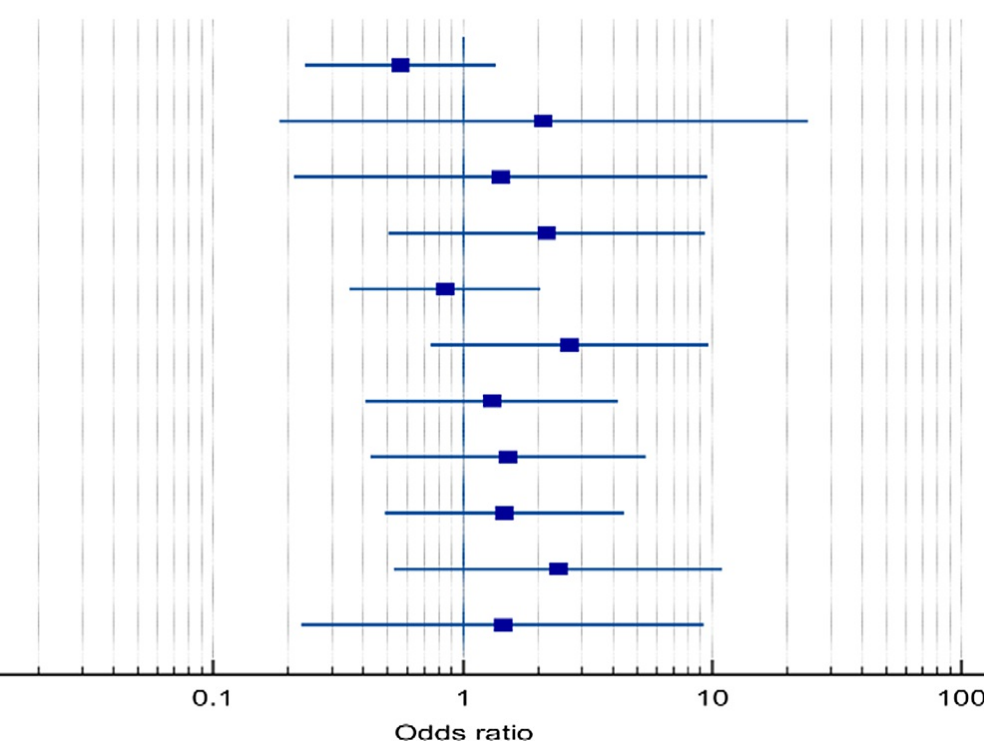
Forest plot for the analyzed tenotomy rate between the standard and accelerated Ponseti methods. A plot representation of the analyzed differences in tenotomy rate between the standard and accelerated Ponseti methods.

Furthermore, 11 studies were included in the meta-analysis to assess the difference between standard and accelerated Ponseti considering the rate of tenotomy. No significant difference was found between the two groups in the tenotomy rate (66.2% in accelerated compared with 63.1% in standard Ponseti, p=0.245). The pooled odds of ratio showed that accelerated method was associated with a slightly higher rate of tenotomy than standard method (OR=1.246, 95%CI: 0.86-1.80, p=0.245; Table 9 and Figure 5).

**Table 9:** Forest analysis of the tenotomy rate differences between accelerated and standard Ponseti methods. Meta-analysis of 11 studies to assess the difference between standard and accelerated Ponseti methods considering the tenotomy rate. Heterogeneity: chi^2^=6.94, df=10 (p=0.731).

Studies	Intervention (accelerated) event/total	Controls (standard) event/total	Weight %	Odds ratio (95 % CI)
Anikwe et al. [25]	12/42	20/48	18.45	0.560 (0.23-1.35)
Doski and Jamal [24]	39/40	37/39	2.41	2.108 (0.18-24.24)
Singh et al. [19]	17/19	18/21	3.94	1.417 (0.21-9.54)
Ahmad et al. [27]	37/40	34/40	6.71	2.176 (0.50-9.39)
Islam et al. [22]	65/77	70/81	18.32	0.851 (0.34-2.06)
Kumar and Singh [21]	9/35	4/35	8.65	2.683 (0.74-9.73)
Dutta et al. [23]	46/52	41/48	10.50	1.309 (0.41-4.21)
Solanki et al. [7]	13/20	11/20	8.85	1.519 (0.43-5.43)
Barik et al. [1]	13/25	11/26	11.75	1.477 (0.49-4.46)
Mageshwaran et al. [18]	6/25	3/26	6.27	2.421 (0.53-10.99)
Elgohary and Abulsaad [17]	30/32	31/34	4.15	1.452 (0.23-9.31)
Total (fixed effects)	287/407	280/418	P=0.245	1.246 (0.86-1.80)

**Figure 6:**
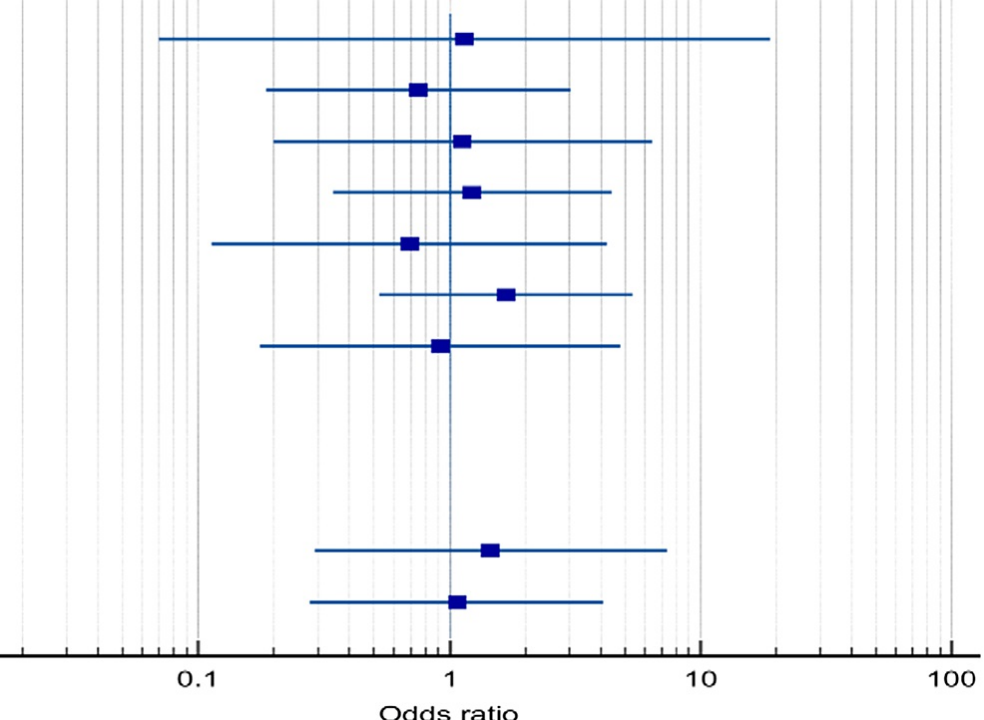
Forest plot for the analyzed relapse rate in the accelerated and standard Ponseti methods. A plot representation of the analyzed relapse rate between patients treated with accelerated and standard Ponseti methods (nine studies are included).

Considering relapse rates reported in both methods, nine studies were included in the meta-analysis. The pooled relapse rate in patients treated with the accelerated Ponseti method was 9.51% compared with 8.54% in patients treated with the standard method. The accelerated method had slightly higher pooled odds of having relapse than the standard method; however, this is not significant (OR=1.126, 95% CI: 0.68-1.86, p=0.642; Table 10 and Figure 6).

**Table 10:** Forest analysis for relapse rate between the standard and accelerated Ponseti methods. Meta-analysis of the relapse rate between patients treated with accelerated and standard Ponseti methods. Heterogeneity: chi^2^=1.22, df=8 (p=0.996).

Studies	Intervention (accelerated) event/total	Controls (standard) event/total	Weight (%)	Odds ratio (95% CI)
Anikwe et al. [25]	1/42	1/48	3.23	1.146 (0.07-18.91)
Doski and Jamal [24]	4/40	5/39	13.04	0.756 (0.19-3.05)
Singh et al. [19]	3/19	3/21	8.43	1.125 (0.19-6.38)
Ahmad et al. [27]	6/40	5/40	15.58	1.235 (0.34-4.43)
Islam et al. [22]	2/77	3/81	7.70	0.693 (0.11-4.27)
Kumar and Singh [21]	9/35	6/35	18.86	1.673 (0.524-5.34)
Dutta et al. [23]	3/52	3/48	9.33	0.918 (0.18-4.78)
Mageshwaran et al. [18]	4/25	3/26	9.81	1.460 (0.29-7.30)
Elgohary and Abulsaad [17]	5/32	5/34	14.03	1.074 (0.28-4.13)
Total (fixed effects)	37/434	34/444	P=0.642	1.126 (0.683-1.86)

**Figure 7:**
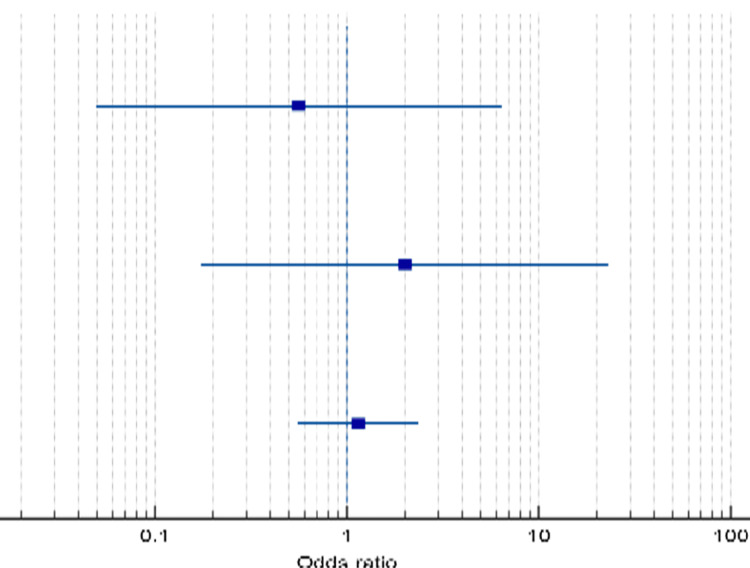
Forest plot of the analyzed complication rate between the standard and accelerated Ponseti methods. A plot representation of the analyzed complication rates between the patients' groups underwent the standard and accelerated Ponseti methods.

Only three studies reported data considering the complication rate of standard and accelerated Ponseti, where the mean complication rate was slightly higher in the accelerated group with rate of 14.4% compared with 13.1% in the standard group; however, no significant difference was reported (OR=1.130, 95% CI: 0.58-2.19, p=0.717; Table 11 and Figure 7).

**Table 11:** Analysis of the complication rate between accelerated and standard Ponseti methods. Meta-analysis of the complication rates between the patients' groups underwent the standard and accelerated Ponseti methods (only three studies are included in this analysis). Heterogeneity: chi^2^=0.528, df=2 (p=0.768).

Studies	Intervention (accelerated) event/total	Controls (standard) event/total	Weight (%)	Odds ratio (95% CI)
Anikwe et al. [25]	1/42	2/48	7.50	0.561 (0.05-6.42)
Doski and Jamal [24]	2/40	1/39	7.47	2.00 (0.17-22.99)
Islam et al. [22]	20/77	19/81	85.04	1.145 (0.56-2.36)
Total (fixed effects)	23/159	22/168	P=0.717	1.130 (0.58-2.19)

Discussion

For the normal development and conservation of the foot function in patients with clubfoot, an early correction of all aspects of the deformity is significant. The Ponseti technique is considered the golden standard for correction of the clubfoot deformity [16,28]. Regarding the psychological and economic aspects of the treatment for the family of patients with CTEV who must travel several times long distances to the treatment center, it has become a necessity to shorten the time needed for treatment for the convenience of both the patients and their parents [2]. However, it is important to ensure that the accelerated Ponseti method has the same effectiveness as the standard method with similar or even lower complications and relapse rates. In this study, we systematically reviewed 13 studies aimed at comparing the two methods, and then we conducted a meta-analysis between variables to obtain pooled results.

The current meta-analysis confirmed the previous studies that showed that the accelerated Ponseti method is associated with a significantly reduced duration of treatment with a mean difference of 19.2 days less than standard Ponseti. In a previous meta-analysis conducted by Savio and Maharjana, the authors reported that the accelerated Ponseti method was superior to the standard method regarding the duration of treatment with 24.25 days compared with 41.54 days, p=0.0001 [13]. The shorter duration of the treatment could be a significant solution for the low compliance of patients and their parents who must travel longer distances for treatment which may result in the treatment's failure.

The accelerated Ponseti casting could be a solution that would allow patients to stay in local accommodation for a shorter time instead of traveling long distances frequently, which would reduce their overall financial burden and associated with improvement in the patient’s compliance, and maximize functional improvement [7,29].

The Pirani scoring system is one of the methods most commonly used to assess clubfoot deformity severity which consists of six components as follows: posterior crease, emptiness of the heel, equinus rigidity, medial crease, curvature of the lateral border of the foot, and reducibility of the lateral talar head [30]. Each component is given a score of zero for no abnormality, 0.5 for moderate abnormality, or 1.0 for severe abnormality and summed for a total score ranging between zero and six where a higher score indicates more severe deformity [31,32]. Based on the results of the current meta-analysis, we found that accelerated Ponseti casting can achieve a comparable Pirani score indicating a more significant reduction in the deformity severity when compared with standard Ponseti casting in agreement with previous studies [13,33].

Another advantage of accelerated Ponseti that is reported in the literature review is that practitioners should note that the accelerated approach is associated with reduced risk for osteopenia and pressure sores, which commonly result from prolonged casting. Although these clubfoot treatment complications mostly resolve naturally within a few months after the end of the treatment, osteopenia has been reported after immobilization in above-knee plasters. Therefore, patients may benefit from the overall shorter duration of treatment reported with the accelerated casting method to avoid or limit the harm from these conditions [34,35]. Other common complications caused by prolonged casting include pressure sores, skin rashes, and disuse atrophy which could be minimized by the accelerated protocol [1]. The accelerated method allows the practitioners to more efficiently monitor these complications and therefore better manage them [34]. However, the results of this meta-analysis showed no significant difference regarding the prevalence of complications between patients who underwent accelerated and standard Ponseti methods with a slightly higher incidence of complications in the accelerated Ponseti method-treated group.

Before the hindfoot is corrected to the natural position, tenotomy is sometimes required to unlock the ossicles from beneath the talus when full dorsiflexion is not possible with stretching alone. Among those cases, further stretching and casting are performed afterward to achieve the complete correction of the deformities [36]. In our study, the rate of tenotomy does not differ significantly between the two procedures, with a slightly higher rate in the accelerated Ponseti method. This could be because of the slightly higher baseline Pirani score in the accelerated group [18] as well as the difference in the severity of deformity or technical error in casting [17].

Moreover, the results of the current study showed comparable relapse rates between the two methods. This has been also reported in some previous studies [8,9,13]. Some studies have linked this outcome to bracing compliance and the level of education of patients' families. Relapse risk can be reduced or prevented by stressing the importance of bracing to family members during the regular follow-up, while clearly teaching them how to correctly fit the orthotics and supervising their initial attempts would help in reducing the relapse rate [16,18,37].

This study had some limitations, including that some of the analyses had high study heterogeneity, especially for Pirani score and number of casts needed. Moreover, most of the studies were conducted in Asia and Africa with no studies conducted in developed countries; however, the extensive research did not identify these settings. On the other hand, this study had some advantages including being the first meta-analysis using recent studies (2015-2022) regarding the comparison between accelerated and standard Ponseti methods. Moreover, all studies included in this review were controlled trials and prospective comparative designs which increased the accuracy and reliability of the meta-analysis.

## Conclusions

The present meta-analysis supports the published conclusions that the accelerated Ponseti method can achieve efficacy comparable to the standard method in terms of post-procedure Pirani score, tenotomy rate, relapse rate, complications rate, and number of casts needed by the patients, with the advantage of requiring shorter duration of treatment, which is expected to achieve more patient’s compliance.

## References

[1] Barik S, Nazeer M, Mani BT (2019). Accelerated Ponseti technique: efficacy in the management of CTEV. Eur J Orthop Surg Traumatol.

[2] Dobbs MB, Gurnett CA (2009). Update on clubfoot: etiology and treatment. Clin Orthop Relat Res.

[3] Ganesan B, Luximon A, Al-Jumaily A, Balasankar SK, Naik GR (2017). Ponseti method in the management of clubfoot under 2 years of age: a systematic review. PLoS One.

[4] Ponseti IV (1996). Congenital Clubfoot: Fundamentals of Treatment. OXFORD Univ Press.

[5] McKay DW (1982). New concept of and approach to clubfoot treatment: section I-principles and morbid anatomy. J Pediatr Orthop.

[6] Fletcher C (2017). The neglected clubfoot. Glob J Med Res.

[7] Mahendra S, Anand A, Sanjay R (2018). Comparative study of accelerated Ponseti method versus standard Ponseti method for the treatment of idiopathic clubfoot. J Orthop Traumatol Rehabil.

[8] Švehlík M, Floh U, Steinwender G, Sperl M, Novak M, Kraus T (2017). Ponseti method is superior to surgical treatment in clubfoot - long-term, randomized, prospective trial. Gait Posture.

[9] Dobbs MB, Nunley R, Schoenecker PL (2006). Long-term follow-up of patients with clubfeet treated with extensive soft-tissue release. J Bone Joint Surg Am.

[10] Turco VJ (1971). Surgical correction of the resistant club foot. One-stage posteromedial release with internal fixation: a preliminary report. J Bone Joint Surg Am.

[11] McKay DW (1983). New concept of and approach to clubfoot treatment: section II - correction of the clubfoot. J Pediatr Orthop.

[12] Herzenberg JE, Radler C, Bor N (2002). Ponseti versus traditional methods of casting for idiopathic clubfoot. J Pediatr Orthop.

[13] Savio SD, Maharjana MA (2021). Accelerated versus standard Ponseti method for idiopathic congenital talipes equinovarus: a systematic review and meta-analysis. Pediatr Traumatol Orthop Reconstr Surg.

[14] Laaveg SJ, Ponseti IV (1980). Long-term results of treatment of congenital club foot. J Bone Joint Surg Am.

[15] Cooper DM, Dietz FR (1995). Treatment of idiopathic clubfoot. A thirty-year follow-up note. J Bone Joint Surg Am.

[16] Cosma DI, Vasilescu DE (2014). Ponseti treatment for clubfoot in Romania: a 9-year single-centre experience. J Pediatr Orthop B.

[17] Elgohary HS, Abulsaad M (2015). Traditional and accelerated Ponseti technique: a comparative study. Eur J Orthop Surg Traumatol.

[18] Mageshwaran S, Murali VK, Devendran R, Yoosuf A, Anandan H (2016). Evaluation of outcome of correction of clubfoot by conventional Ponseti and accelerated Ponseti. Int J Sci Study.

[19] Singh PV, Ghani A, Singh T, Malik AT, Singh S (2021). Comparative analysis of correction of idiopathic congenital talipes equinovarus by conventional and accelerated Ponseti method with minimum 12 months follow up in a tertiary care hospital in North India. Int J Res Med Sci.

[20] Sharma P, Yadav V, Verma R, Gohiya A, Gaur S (2018). Comparative analysis of results between conventional and accelerated Ponseti technique for idiopathic congenital clubfoot. Orthop J MP Chap.

[21] Kumar R, Singh SK (2020). Conventional Ponseti vs accelerated Ponseti in the management of cases of idiopathic clubfoot. Eur J Mol Clin Med.

[22] Islam MS, Masood QM, Bashir A, Shah FY, Halwai MA (2020). Results of a standard versus an accelerated Ponseti protocol for clubfoot: a prospective randomized study. Clin Orthop Surg.

[23] Dutta A, Sipani AK, Jain PK (2019). A comparative study between standard and accelerated Ponseti method in management of idiopathic congenital talipes equinovarus. Int J Orthop Sci.

[24] Doski JO, Jamal IB (2021). Accelerated versus conventional Ponseti protocol for the treatment of idiopathic talipes equinovarus deformity: a short term follow up in Iraq. Zanco J Med Sci.

[25] Anikwe A, Lasebikan OA, Enweani UN. (2021). Comparison of standard and accelerated Ponseti technique in the treatment of idiopathic clubfoot at a regional orthopaedic hospital in Nigeria. Int J Paediatr Orthop.

[26] Ahmed J, Shahid S, Alam W (2019). Outcome of patients suffering from congenital idiopathic club foot: a comparative analysis of using classical versus accelerated Ponseti techniques. J Pak Orthop Assoc.

[27] Ahmad DMN (2020). Comparative study of accelerated Ponseti cast with standard Ponseti cast. J Med Sci Clin Res.

[28] Hennessey TA (2012). Congenital clubfoot and the Ponseti method. Curr Orthop Pract.

[29] Herring J (2020). Tachdjian’s Pediatric Orthopaedics: From the Texas Scottish Rite Hospital for Children. Sixth Edition. Tachdjian’s Pediatric Orthopaedics: From the Texas Scottish Rite Hospital for Children, 6th Edition. 7 edition. © Elsevier.

[30] Mejabi J, Esan O, Adegbehingbe O (2016). The Pirani scoring system is effective in assessing severity and monitoring treatment of clubfeet in children. Br J Med Med Res.

[31] Lampasi M, Abati CN, Bettuzzi C, Stilli S, Trisolino G (2018). Comparison of Dimeglio and Pirani score in predicting number of casts and need for tenotomy in clubfoot correction using the Ponseti method. Int Orthop.

[32] Dyer PJ, Davis N (2006). The role of the Pirani scoring system in the management of club foot by the Ponseti method. J Bone Joint Surg Br.

[33] Harnett P, Freeman R, Harrison WJ, Brown LC, Beckles V (2011). An accelerated Ponseti versus the standard Ponseti method: a prospective randomised controlled trial. J Bone Joint Surg Br.

[34] Sahu B, Rajavelu R, Tudu B (2015). Management of idiopathic congenital talipes equinovarus by standard versus accelerated Ponseti plaster technique: a prospective study. J Orthop Traumatol Rehabil.

[35] Lourenço AF, Morcuende JA (2007). Correction of neglected idiopathic club foot by the Ponseti method. J Bone Joint Surg Br.

[36] Goriainov V, Judd J, Uglow M (2010). Does the Pirani score predict relapse in clubfoot?. J Child Orthop.

[37] Rahman MS, Alam MK, Shahiduzzaman M (2015). Percutaneous needle tenotomy for Ponseti technique in the management of Congenital Talipes Equinovarus (CTEV). J Dhaka Med Coll.

